# Natural ^15^N abundance in specific amino acids indicates associations between transamination rates and residual feed intake in beef cattle

**DOI:** 10.1093/jas/skaa171

**Published:** 2020-05-20

**Authors:** Gonzalo Cantalapiedra-Hijar, Pablo Guarnido, Anne-Marie Schiphorst, Richard J Robins, Gilles Renand, Isabelle Ortigues-Marty

**Affiliations:** 1 INRAE, Université Clermont Auvergne, Vetagro Sup, UMRH, Saint-Genès-Champanelle, France; 2 Université de Nantes, CNRS, CEISAM UMR, Nantes, France; 3 Université Paris-Saclay, INRAE, AgroParisTech, GABI, Jouy-en-Josas, France

**Keywords:** amino acid transamination, beef cattle, nitrogen isotopes, residual feed intake

## Abstract

Improving the ability of animals to convert feed resources into food for humans is needed for more sustainable livestock systems. Genetic selection for animals eating less while maintaining their performance (i.e., low residual feed intake [**RFI**]) appears a smart strategy but its effectiveness relies on high-throughput animal phenotyping. Here, we explored plasma nitrogen (N) isotope ratios in an attempt to identify easily superior young bulls in terms of RFI. For this, 48 Charolais young bulls fed two contrasting diets (corn vs. grass silage diets) were selected from a larger population as extreme RFI animals (24 low-RFI vs. 24 high-RFI) and their plasma analyzed for natural ^15^N abundance (**δ**^**15**^**N**) in the whole protein (bulk protein) and in the individual protein-bound amino acids (**PbAA**). For the first time, we showed that the δ ^15^N in plasma bulk protein differed (*P* = 0.007) between efficient (low-RFI) and inefficient (high-RFI) cattle regardless of diet. Furthermore, most analyzed PbAA followed the same trend as the bulk protein, with lower (*P* < 0.05) δ ^15^N values in more efficient (low-RFI) compared with less efficient (high-RFI) cattle, again regardless of diet. The only three exceptions were Phe, Met, and Lys (*P* > 0.05) for which the first metabolic reaction before being catabolized does not involve transamination, a pathway known naturally to enrich AAs in ^15^N. The contrasted isotopic signatures across RFI groups only in those PbAA undergoing transamination are interpreted as differences in transamination rates and N-use efficiency between low- and high-RFI phenotypes. Natural isotopic N signatures in bulk proteins and specific PbAA can be proposed as biomarkers of RFI in growing beef cattle fed different diets. However, the current study cannot delineate whether this effect only occurs post-absorption or to some extent also in the rumen. Our data support the conclusion that most efficient cattle in terms of RFI upregulate N conservation mechanisms compared with less efficient cattle and justify future research on this topic.

## Introduction

In the current context of a growing global demand for animal products, the efficiency by which feed resources are used for animal production (i.e., feed efficiency [**FE]**) becomes a key issue for sustainable livestock systems (Mottet et al., 2017). Beyond the feeding strategy, decreasing animal feed intake while maintaining animal performance is a current genetic selection goal which potentially impacts on the profitability and sustainability of the beef cattle industry (Hill, 2012). Residual feed intake (**RFI**)—defined as the difference between the animal’s actual feed intake and that theoretically expected from observed animal performance (Koch et al., 1963)—is currently the most worldwide used index for FE selection. Because determining RFI is expensive and time-consuming, needing at least 10 wk of individual intake and growth recording (Herd et al., 2003), several investigations have explored biomarkers of RFI (Karisa et al., 2014; Meale et al., 2017). However, none of these have been validated and accepted so far, probably because of the difficulty of identifying biological markers reflecting processes driven by the inter-individual variability rather than by dietary treatments. Given the biological closeness between FE and the animal’s ability to convert feed nitrogen (N) into animal protein (i.e., N-use efficiency) in growing cattle (Cantalapiedra-Hijar et al., 2015), one promising approach to predict differences in RFI could be focusing on metabolites and proxies reflecting changes in N partitioning and protein turnover across individuals (Elolimy et al., 2019).

The natural ^15^N abundance (**δ**^**15**^**N**) of animal proteins is usually higher than the δ ^15^N of the consumed diet (DeNiro and Epstein, 1981), the so-called isotopic N discrimination (**Δ**^**15**^**N** = δ ^15^N_animal_ − δ ^15^N_diet_). The Δ ^15^N is the result of isotopic fractionation processes (i.e., enrichment of one isotope relative to another in a chemical or physical process) occurring during N transfer from feed to animal tissues (Cantalapiedra-Hijar et al., 2016; Nasrollahi et al., 2020). The Δ ^15^N is relatively variable across animals and diets, ranging from 0.1% to 6.4‰ (Cantalapiedra-Hijar et al., 2018). This variability in Δ ^15^N has recently been linked to the between-animal variability in N-use efficiency (Cantalapiedra-Hijar et al., 2018). However, when this isotopic biomarker was applied to RFI, no significant relationships were obtained (Wheadon et al., 2014; Meale et al., 2017). These results were unexpected because more efficient growing individuals, based on their RFI values, eat less N while having similar protein retention (Lines et al., 2014; de Assis Lage et al., 2019) and this should translate into a higher N-use efficiency.

The isotopic signature of whole protein (bulk protein) represents the weight-averaged isotopic signatures of their amino acid (**AA**) composition, which may differ across individual AAs depending on their specific post-absorptive catabolic pathways (Braun et al., 2014; O’Connell, 2017) and, probably, to a minor extent on their biosynthetic routes in bacteria (Macko and Estep, 1984). In particular, the isotopic N signatures of those AAs more prone to cycle their amino-N with others through reversible transamination appear to show greater natural ^15^N enrichment compared with those lacking this ability (McMahon and McCarthy, 2016; O’Connell, 2017). Therefore, the N isotope composition of individual AAs in animal proteins carries different metabolic information (Braun et al., 2014) and might give deeper insight into the protein metabolism of organisms (O’Connell, 2017). Our hypothesis is that the expected lower AA catabolism in more efficient cattle would translate into a lower isotopic ^15^N enrichment of only those specific AAs able to undergo reversible transamination before being catabolized. Hence, the objective of the present study was to explore to what extent the analysis of individual protein-bound AA (**PbAA**) would discriminate phenotypically extreme animals according to their RFI compared with the same analysis in N of bulk plasma proteins and to assess if conclusions based on this comparison depended on the type of diet.

## Materials and Methods

The experiment was conducted in compliance with the French Legislation on Animal Care. The C2EA-02 Animal Research Ethics Committee (Auvergne, France) prospectively approved this research and thereafter the Ministry of Agriculture (France) validated it with the approval number APAFIS#2930-2015111814299194v3.

### Feed efficiency test

This study is lodged within a large program aiming to explore and validate biomarkers of RFI. This is comprised of 364 purebred Charolais bulls (380 ± 58 kg body weight and 301 ± 15 d old at the onset of the experiment) at three different experimental farms belonging to the French Chamber of Agriculture. All animals were offspring by insemination of 58 Charolais bulls and were housed indoors. These animals were tested for RFI between 2015 and 2018, leading to seven independent cohorts (farm × period). Each cohort contained between 48 and 63 animals allotted into pens of 5 to 8 animals each, according to their body weight, age, and genetic origin. Animals within each cohort were evenly assigned to either a high-starch corn silage diet or a high-fiber grass silage diet (E-Supplementary Table S1). Diet formulation within dietary treatments was almost constant across cohorts with only minor changes in feed proportions to take into account time and location variability in silages chemical composition. Both diets were formulated (see E-Supplementary Table S1) with a forage to concentrate ratio close to 65:35 and with a metabolizable protein to net energy ratio close to recommendations (INRA, 2018). Animals were adapted to their assigned experimental diet for at least 4 wk, after which an RFI test started for 180 to 226 d according to the cohort. Daily dry matter intake (**DMI**) was individually recorded throughout the experiment using an automatic intake recoding system based on troughs placed on weighing cells (Biocontrol, Rakkestad, Norway). Body weight was recorded every 2 wk at the same time (2:00 p.m.). The average daily gain (**ADG**) of each animal was calculated as the slope of body weight (recorded fortnightly) regressed against time. For calculating RFI, the observed DMI was predicted from the ADG, mean metabolic body weight, and the effect of contemporary group, the latter defined at the level of pen within each cohort × diet. The obtained model explained 86% of the DMI variance, the obtained standard deviation for RFI was 0.435 kg/d, and all the included effects were significant at *P* < 0.001. To maximize the chance of finding RFI biomarkers and based on previous experience showing the strong cohort effect on plasma metabolites (Meale et al., 2017), 48 extreme animals (24 low-RFI and 24 high-RFI) were selected from the 364 available animals taking into account three criteria: 1) minimum number of selected cohorts (avoiding experimental variability unrelated to RFI), 2) maximal RFI contrast, and 3) balanced number of animals per condition (diet, cohort, and RFI group). Consequently, the selected 48 extreme-RFI bulls belonged to only 3 out of 7 different available cohorts (167 out of 364 animals), and, for each of these 3 cohorts, the 4 lowest and highest RFI animals were selected within each diet (16 extreme RFI animals per cohort). Selected animals differed in RFI by 1.48 kg/d on average (1.42 and 1.55 kg/d for corn and grass diets, respectively). Blood was sampled from each animal by coccygeal venipuncture (9 mL heparinized tubes, BD vacutainer, Plymouth, UK) before the morning meal distribution and 1 mo before the end of their RFI test (corresponding to an age of 17.2 ± 0.51 mo). Blood was centrifuged within the 15 min after sampling (10 min, 2,500 × *g*, 4 °C) and plasma was stored at −80 °C until used for isotopic analysis.

### Laboratory isotopic analysis

Bulk plasma proteins were isolated from 750 μL of heparinized plasma by precipitation with 37.5 μL of sulfosalicylic acid solution (1 g/mL). Following centrifugation (2,500 × *g*; 15 min) and washing (distilled water), the pellet was freeze-dried. Nitrogen isotopic composition (δ ^15^N; ‰) was determined on freeze-dried plasma proteins (bulk protein) using an isotope-ratio mass spectrometer (irms; Isoprime, VG Instruments, Manchester, UK) coupled to an elemental analyzer (EA; Isoprime, VG Instruments, Manchester, UK). Each sample was analyzed in duplicate for bulk protein with intra-sample coefficient of variation (CV) of 2%. Results were expressed using the delta notation in parts per 1,000 (‰) related to the international standard, atmospheric N_2_ (*R*_standard_ = 0.0036765), via the in-house standard glutamic acid (run every 10 samples).

Sample preparation and isotope analysis of PbAA released from plasma proteins were carried out as described previously (Meale et al., 2017). Briefly, following derivatization of AAs released by acid hydrolysis of plasma proteins (37% HCl; 24 h; 105 °C), N isotopic ratios were measured using a Delta V Advantage isotope ratio mass spectrometer coupled to a Trace Ultra gas chromatograph **(GC)** via a GC combustion Interface III (Thermo Fisher Scientific, Bremen, Germany). The mass spectrometer was calibrated for δ ^15^N(‰) values by reference to pulses of pure N_2_ gas from a cylinder calibrated by the elemental analyzer (EA-irms) against international reference materials IAEA-N1 or IAEA-N2 (IAEA, Vienna, Austria). Values of δ ^15^N for each AA were measured by GC-irms and the output data normalized using a correction factor representing the differences in δ ^15^N values obtained for pure AA between EA-irms and GC-irms. Among the 20 standard proteinogenic AAs, we only measured the following: Ala, Gly, Val, Ile + Leu (**Ilx**), Pro, Ser, Glu + Gln (**Glx**), Met, Phe, and Lys. Because Ile and Leu co-eluted, the separation of their peaks was not possible. Thus, the reported δ ^15^N values for Ilx represent the weighted average of these two AAs. Similarly, because during acid hydrolysis Gln is deaminated to Glu, our δ ^15^N values for Glx represent a weighted average of these two AAs. Samples were analyzed in triplicate with intra-sample CV values ranging from 2% (Pro, Glx) to 8% (Met). The checking process of each step of the irms-GC/MS procedure belongs to a quality assurance process to give useable results. The use of several standards to verify irms repeatability and accuracy was verified by pure N_2_ gas pulses and international reference. GC efficiency and GC Combustion interface III efficiency were tested by measuring a mix of commercial AA standards. This analysis verified chromatographic resolution and repeatability of the combustion/reduction step. This standard mix contains the same 12 AAs that are well separated as their N-pivaloyl-*iso*-propyl (**NPIP**) esters. Some AAs are not derivatized by NPIP and, therefore, were not quantified and reported in this study. To verify the efficiency of NPIP derivatization, an internal standard (norleucine) is introduced in each sample before derivatization, so that this internal standard undergoes the same treatment from preparation to results.

### Statistical analysis

Performance data and isotopic values were analyzed by ANOVA in R software (R Development Core Team, 2009) taking into account the fixed effect of cohort (*n* = 1, 2, 3), diet (corn vs. grass), RFI group (low vs. high RFI), and their interactions. Significant effects were declared at *P* ≤ 0.05.

## Results

**Figure 1.**
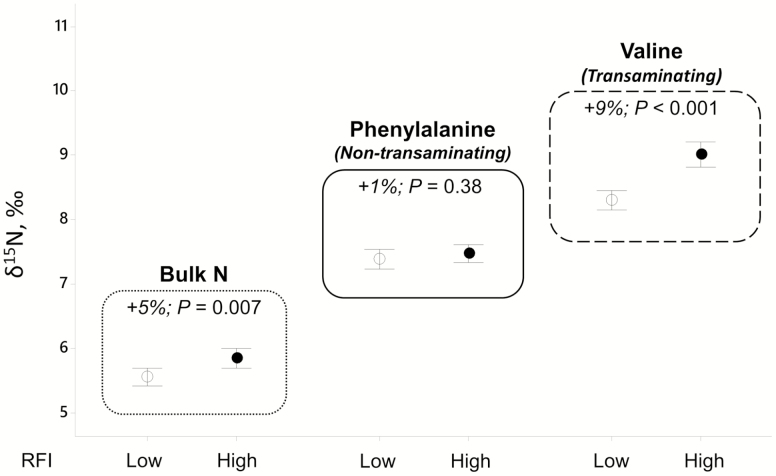
Natural ^15^N abundances (δ ^15^N, ‰) in total plasma proteins (bulk N) and plasma protein-bound phenylalanine (representing the non-transaminating amino acid) and valine (representing the transaminating amino acid) in young Charolais bulls showing extreme RFI. Confidence intervals (95%) are presented with values adjusted for the effect of cohort, diet, and their interactions. Change (%) with associated *P*-values refers to the isotopic difference between low and high RFI groups.

The 48 selected extreme animals showed contrasted RFI values (*P* < 0.001) with inefficient (high-RFI) animals eating on average 1.29 kg dry matter/d more (+16%; *P* < 0.001) than their efficient (low-RFI) counterparts (Table 1) at similar ADG (*P* = 0.44). Consequently, the feed conversion efficiency was greater (+18%; *P* <0.001) in low- compared with high-RFI animals. In this context, the δ ^15^N values measured in plasma proteins (bulk protein) were significantly affected by the RFI group with higher δ ^15^N values in high- vs. low-RFI animals (+5%; *P* = 0.007). When the same isotopic analysis was conducted on PbAA released from plasma protein, rather than the bulk protein, the effect of RFI group on natural ^15^N abundance strongly depended on the individual AA (Figure 1). Indeed, as shown in Table 1, whereas the δ ^15^N values of most PbAA measured in this study (Ala, Gly, Val, Ilx, Pro, Ser, and Glx) were significantly higher in inefficient (high RFI) compared with efficient (low RFI) animals (average change of +9%; *P* ≤ 0.001), others were not significantly affected, notably Phe (+1%; *P* = 0.60) and Lys (average change +4%; *P* = 0.39), or tended to be higher only in grass silage diets, notably Met (Diet × RFI interaction; *P* = 0.09). The average δ ^15^N values of all PbAA were higher in inefficient (high-RFI) than efficient (low-RFI) animals (+7%; *P* < 0.001). Furthermore, diet significantly (*P* ≤ 0.03) affected the δ ^15^N values of both bulk protein and all PbAA. Moreover, because the chemical composition of diets slightly differed across cohorts (E-Supplementary Table S1), the interaction Cohort × Diet on δ ^15^N values was significant for all PbAA (*P* < 0.05; data not shown) except for Met (*P* = 0.27).

**Table 1.** Animal performance criteria and natural ^15^N abundances in total plasma proteins (δ ^15^N-bulk) and plasma PbAA (δ ^15^N-PbAA) of young Charolais bulls showing extreme RFI when fed either a corn silage- or a grass silage-based diet

	Corn silage diet^1^		Grass silage diet^1^			*P*-value		
	Low RFI	High RFI	Low RFI	High RFI	SEM	DIET	RFI	DIET × RFI
No. of animals	12	12	12	12				
RFI, kg/d	−0.71	0.70	−0.78	0.77	0.064	0.96	<0.001	0.26
ADG, kg/d	1.56	1.63	1.49	1.33	0.058	0.003	0.44	0.08
DMI, kg/d	8.92	10.6	8.69	9.59	0.267	0.03	<0.001	0.17
FCE^2^, kg/kg	0.175	0.154	0.171	0.138	0.0035	0.008	<0.001	0.12
δ ^15^N-Bulk, ‰	5.18	5.48	5.93	6.21	0.101	<0.001	0.007	0.92
δ ^15^N-PbAA, ‰								
Ala	9.07	9.59	9.43	10.5	0.159	<0.001	<0.001	0.11
Gly	7.39	8.12	7.95	8.44	0.172	0.01	0.001	0.55
Val	7.88	8.58	8.72	9.44	0.127	<0.001	<0.001	0.91
Ilx	5.37	5.92	5.99	6.75	0.138	<0.001	<0.001	0.47
Pro	10.2	11.0	11.5	11.9	0.144	<0.001	<0.001	0.14
Ser	5.76	6.42	6.18	6.82	0.178	0.03	<0.001	0.97
Glx	6.93	7.61	7.98	8.39	0.142	<0.001	<0.001	0.35
Met	4.96	4.97	5.58	6.33	0.215	<0.001	0.08	0.09
Phe	6.26	6.20	8.50	8.74	0.106	<0.001	0.38	0.18
Lys	2.71	2.85	4.01	4.12	0.128	<0.001	0.33	0.86
Total^3^	6.65	7.13	7.58	8.15	0.087	<0.001	<0.001	0.62

^1^Data are averaged for the three cohorts.

^2^Feed conversion efficiency calculated as ADG divided by DMI.

^3^Average δ ^15^N values from analyzed AA.

## Discussion

The contrasting N isotopic signatures observed in the present study across RFI groups for some specific PbAA (Ala, Gly, Val, Ile + Leu, Pro, Ser, Glx) but not for others (Phe, Met, Lys) are interpreted as indicating differences in AA transamination rates that will be related to differences in N-use efficiency between low- and high-RFI phenotypes. Before being catabolized, many, but not all, AAs undergo as first reaction either a transamination (i.e., the reversible transfer of an amino group from a donor AA to an acceptor ketoacid) or a deamination (i.e., the removal of an amino group from an AA). Enzymes participating in AA transamination and deamination have been shown to have kinetic isotope effects, which favor reaction with AA isotopomers containing the lighter N isotope (^14^N) over the heavier one (^15^N). This isotopic preference occurs because cleaving the C-N bond of an AA requires less energy and is, therefore, faster when it contains the lighter isotope of N (Macko et al., 1986; Miura and Goto, 2012). Because the catabolic pathways vary across individual AAs, their N isotope signatures carry different metabolic information (Braun et al., 2014; O’Connell, 2017). For most of those PbAA herein analyzed and showing lower δ ^15^N values in efficient (low-RFI) than in inefficient (high-RFI) animals, it is known that their amino-N group is readily interchangeable with the metabolic N pool (Ala, Val, Ilx, Pro, Glx; called “trophic” AAs in O’Connell, 2017) or may be exchanged with other AAs by reversible transamination (Gly and Ser: Braun et al., 2014; McMahon and McCarthy, 2016). Conversely, the only three PbAA herein analyzed not showing differences across RFI groups belong to those AAs not undergoing transamination as an initial step in their catabolism (Phe, Met, and Lys; called “source” AAs in O’Connell, 2017). The dominant initial step of Phe catabolism in mammals does not involve transamination but irreversible hydroxylation to Tyr catalyzed by phenylalanine monooxidase. This reaction neither forms nor breaks C-N bonds and thus is not associated with enzymatic isotopic fractionation (Chikaraishi et al., 2007; O’Connell, 2017). The formed Tyr then undergoes transamination with the N transferred to α-ketoglutarate to form Glu before eventually entering the urea cycle. This is probably why Tyr, not measured in this study, usually has higher ^15^N values compared with Phe both in fish and in mammals (see meta-analysis by McMahon and McCarthy, 2016) as well as in ruminant animals (Cantalapiedra-Hijar et al., 2016). Lys is atypical, in that it cannot be formed from its corresponding α-ketoacid as can most AAs. Rather, the primary pathway of Lys catabolism involves the formation of saccharopine, which is not formed from transamination but by the condensation of Lys and α-ketoglutarate. Again, no C-N bonds are broken during this catabolic pathway. In the third case, Met, the primary metabolic pathway does not involve transamination either. Methionine is generally metabolized through the transmethylation–transsulfuration pathway leading to the formation of S-adenosylmethionine as first intermediate. Because this pathway, together with the “salvage pathway” forming Met from homocysteine, occurs without breaking C-N bonds, little isotopic N fractionation is thus expected (McMahon and McCarthy, 2016; O’Connell, 2017). These special metabolic features of these three AAs (i.e., their primary step in catabolism does not involve transamination) explain their usually weak isotopic ^15^N enrichment in animal tissues relative to the consumed diet (i.e., small isotopic N discrimination; Δ ^15^N). Hence, ecologists have proposed the isotopic signatures of this group of AAs as natural markers of the diet when assessing the consumer–resource relationships within the food web (McMahon and McCarthy, 2016).

It is noteworthy in the present study that, in spite of a systemic dietary effect on δ ^15^N of all analyzed PbAA, those showing the highest differences across diets are indeed these three non-transaminating AAs (Phe, Met, and Lys) having +33% higher ^15^N values in grass vs. corn diets vs. +9% observed in the others. This highlights their acknowledged ability to reflect the isotopic signature of the diet rather than the metabolic changes occurring in the animal (McMahon and McCarthy, 2016; O’Connell, 2017). An exception to this conclusion might be the isotopic N composition of Met when animals are fed grass silage diets since a trend for inefficient animals to have higher δ ^15^N values of Met was observed only in this diet (+13%). It is difficult to explain this finding but given that this specific AA is known to be the first-limiting AA for growth in cattle fed grass-silage diets (Cantalapiedra-Hijar et al., 2020), it could be reasonably hypothesized that differences across animals of Met utilization occurred in this unfavorable nutritional context. While there is a potential transamination pathway for Met via methionine adenosyltransferase (Blom et al. 1989), the primary metabolic pathway of Met involves transsulfuration to other sulfur-containing AAs (Stipanuk, 1986). However, under certain situations, this minor catabolic pathway may become important such as a lowered availability of methyl groups to ensure the re-methylation of homocysteine to methionine (Storch et al, 1988). This transamination pathway related to Met metabolism may involve isotopic N fractionation and be responsible for our observation. More research is warranted to elucidate this interesting finding, opening the door to future biomarkers of AA nutritional status of farm animals.

Unlike Met, Phe, and Lys, those AAs undergoing transamination seem to indicate better the metabolic differences across RFI groups. Furthermore, with grass silage diets, the data do not support the assumed higher ^15^N values in Glu compared with Phe extensively reported in the literature (meta-analysis by McMahon and McCarthy, 2016). Rather, our data confirm once again the proposed potential influence of rumen microbiota in generating the unexpectedly higher δ ^15^N values of Phe (Cantalapiedra-Hijar et al., 2016; Meale et al., 2017). In this sense, our results should be taken with caution when interpreting differences in isotopic N composition across PbAA as a consequence only of animal metabolism, because previous results demonstrated that rumen microbiota are responsible to a variable extent for the isotopic N discrimination between the animal proteins and the diet (Cantalapiedra-Hijar et al., 2016; Nasrollahi et al., 2020 and our current experimental diets promoted a high proportion of absorbed AA from the microbial origin (2/3 on average; E-Supplementary Table S1). Hence, we cannot rule out the possibility of a rumen effect in combination with animal metabolism on the observed differences in δ ^15^N of PbAA across conditions. However, although this rumen effect remains plausible, the relatively high contribution of metabolic processes to explain differences in RFI (see reviews by Richardson and Herd, 2004 and Cantalapiedra-Hijar et al., 2018) suggests that most of the observed differences in δ ^15^N of PbAA across RFI groups have likely a metabolic rather than ruminal origin.

Some results from the literature highlight that AA transamination rates slowdown in situations where organisms try to economize N. In humans, the transamination rate of Leu greatly decreased throughout pregnancy and closely paralleled the fall in urea production (Kalhan et al., 1998). Likewise, a decrease in the Leu transamination rate was observed in insulin-treated diabetic patients concurrently with a decrease in muscle protein breakdown (Meek et al., 1998). In rats, transaminase activities decreased as dietary protein levels decreased (Muramatsu and Ashida, 1962) or when protein restriction led to a fall in blood urea concentration (Brown et al., 1972). In poultry, although not completely consistent across time, a downregulation of different transaminases in four different tissues was observed in efficient (low RFI) compared with inefficient (high RFI) lines (Aggrey et al., 2014). To the best of our knowledge, similar results are lacking in ruminants, where only a reduction in plasma aspartate aminotransferase concentration (activity was not assessed) has been observed in efficient (low RFI) compared with inefficient (high RFI) beef cattle in one study (Richardson et al., 2004). Because, however, changes in transaminase plasma concentration do not reflect transaminase activities but rather liver injury, the results from Richardson et al. (2004) should be treated with caution.

In the present study, the natural ^15^N abundance of bulk plasma proteins (δ ^15^N in bulk protein; Table 1) followed the trend observed for the transaminating AAs, with higher values in inefficient vs. efficient cattle (+5%; *P* = 0.007), regardless of the diet. The isotopic signatures in bulk animal proteins result from the averaged values of the isotopic signatures of their amino acids. This is the first time this isotopic biomarker has been shown to discriminate individuals with extreme RFI phenotypes. Unlike in previous reports (Wheadon et al., 2014; Meale et al., 2017), our 48 young bulls showed large differences in RFI (on average a difference of 1.48 kg DMI/d) because they were specifically selected from a large experimental population (*n* = 364). A recent meta-analysis found that the more efficiently the ruminant assimilates the dietary N the lower the animal proteins are ^15^N enriched over their diet (Cantalapiedra-Hijar et al., 2018). Thus, the present results on δ ^15^N in bulk protein suggest that more efficient young bulls (low-RFI) have a greater N-use efficiency compared with their less efficient (high-RFI) counterparts, supporting previous results where low-RFI growing ruminants had less N intake but retained a similar amount (Lines et al., 2014; de Assis Lage et al., 2019). Moreover, the present data concerning feed conversion efficiency (ADG/DMI) also support a greater N-use efficiency in low- compared with high-RFI animals. Our data are noteworthy in that, for the first time, they point to the mechanism of N conservation in beef cattle differing in RFI and the ability of isotopic N signatures to indicate differences in individual N transfer characteristics. These deductions now need to be confirmed with direct measurements of N partitioning and tissue transaminase activities in RFI divergent ruminants. Further studies are also warranted to elucidate the extent to which rumen bacteria may be contributing to the contrasted N isotopic enrichment of PbAA observed across RFI groups.

## Supplementary Material

skaa171_suppl_Supplementary_Material

## Abbreviations

δ ^15^N: natural 15N abundance
Δ ^15^N: isotopic N discrimination between the animal and the diet (δ ^15^N_animal −_ δ ^15^N_diet_)
AA: amino acid
ADG: average daily gain
DMI: dry matter intake
EA: elemental analyzer
FE: feed efficiency
GC: gas chromatograph
Glx: Glu + Gln
Ilx: Leu + Ile
irms: isotope-ratio mass spectrometer
NPIP: N-pivaloyl-*iso*-propyl
PbAA: individual protein-bound amino acids
RFI: residual feed intake
