# Supplementary material for: Natural 15N abundance in specific amino acids indicates associations between transamination rates and residual feed intake in beef cattle
Source: J Anim Sci. 2020 May 20;98(6):skaa171. doi: 10.1093/jas/skaa171 (PMC7275638; doi:10.1093/jas/skaa171)
Supplement: skaa171_suppl_Supplementary_Material [file skaa171_suppl_supplementary_material.docx]

E-Supplement Table 1. Ingredient, chemical composition and nutritive values of experimental diets tested on fattening Charolais bulls.

|  |  | Diets^1^ | | |
| --- | --- | --- | --- | --- |
|  |  | Corn |  | Grass |
| Ingredient composition, % of dry matter |  |  |  |  |
| Corn silage |  | 61.1 ± 2.2 |  | - |
| Grass silage |  | - |  | 62.9 ± 1.1 |
| Wheat straw |  | 5.22 ± 0.8 |  | 4.75 ± 0.7 |
| Wheat grains |  | 20.9 ± 1.4 |  | 5.75 ± 2.0 |
| Beet pulp |  | - |  | 21.9 ± 2.7 |
| Soybean meal |  | 12.8 ± 0.4 |  | 4.71 ± 2.2 |
| Chemical composition, % of dry matter |  |  |  |  |
| Organic matter |  | 97.0 ± 1.6 |  | 91.5 ± 0.36 |
| Crud protein |  | 14.6 ± 0.25 |  | 13.8 ± 0.57 |
| Neutral detergent fiber |  | 33.5 ± 0.21 |  | 49.9 ± 0.20 |
| Starch |  | 30.1 ± 0.10 |  | 4.1 ± 0.11 |
| Feed Values^2^ |  |  |  |  |
| Net energy, Mcal/kg of dry matter |  | 1.62 ± 0.01 |  | 1.49 ± 0.01 |
| Metabolizable protein, g/kg of dry matter |  | 86.3 ± 0.6 |  | 80.3 ± 2.5 |
| Microbial protein, % |  | 62.2 ± 1.3 |  | 67.0 ± 2.0 |
| Rumen bypass protein, % |  | 37.8 ± 1.3 |  | 33.0 ± 1.3 |
| MP/NE^3^, g/Mcal |  | 51.7 ±1.69 |  | 50.7 ± 0.88 |

^1^Average and standard deviation are presented for diets used in the 3 cohorts analyzed in this experiment.

^2^Feed values were estimated from analyzed chemical composition and INRA2018 equations using Systoolweb v1.2 ([www.systool.fr](http://www.systool.fr))

^3^Metabolizable protein to net energy ratio. Recommended values are between and 53 and 48 for body weights of 300 and 600 kg, respectively, in fattening Charolais bulls (INRA, 2018).
